# A Metric of Societal Burden Based on Virus Succession to Determine Economic Losses and Health Benefits of China’s Lockdown Policies: Model Development and Validation

**DOI:** 10.2196/48043

**Published:** 2024-06-07

**Authors:** Wenxiu Chen, Bin Zhang, Chen Wang, Wei An, Shashika Kumudumali Guruge, Ho-kwong Chui, Min Yang

**Affiliations:** 1 National Engineering Reaserch Center of Industrial Wastewater Detoxification and Resource Recovery Research Center for Eco-Environmental Sciences Chinese Academy of Sciences Beijing China; 2 University of Chinese Academy of Sciences College of Resources and Environment Beijing China; 3 Environmental Protection Department Hong Kong SAR Government Hong Kong China

**Keywords:** SARS-CoV-2, lockdown, virus succession, benefit, loss, fatality rate, pandemic, blanket lockdown, partial lockdown

## Abstract

**Background:**

The COVID-19 pandemic had a profound impact on the global health system and economic structure. Although the implementation of lockdown measures achieved notable success in curbing the spread of the pandemic, it concurrently incurred substantial socioeconomic costs.

**Objective:**

The objective of this study was to delineate an equilibrium between the economic losses and health benefits of lockdown measures, with the aim of identifying the optimal boundary conditions for implementing these measures at various pandemic phases.

**Methods:**

This study used a model to estimate the half-lives of the observed case fatality rates of different strains. It was based on global infection and death data collected by the World Health Organization and strain sequence time series data provided by Nextstrain. The connection between the health benefits and economic losses brought by lockdown measures was established through the calculation of disability-adjusted life years. Taking China’s city lockdowns as an example, this study determined the cost-benefit boundary of various lockdown measures during the evolution of COVID-19.

**Results:**

The study reveals a direct proportionality between economic losses due to lockdowns and the observed case fatality rates of virus strains, a relationship that holds true irrespective of population size or per capita economic output. As SARS-CoV-2 strains evolve and population immunity shifts, there has been a notable decrease in the observed case fatality rate over time, exhibiting a half-life of roughly 8 months. This decline in fatality rates may offset the health benefits of maintaining unchanged lockdown measures, given that the resultant economic losses might exceed the health benefits.

**Conclusions:**

The initial enforcement of lockdown in Wuhan led to significant health benefits. However, with the decline in the observed case fatality rate of the virus strains, the economic losses increasingly outweighed the health benefits. Consequently, it is essential to consistently refine and enhance lockdown strategies in accordance with the evolving fatality and infection rates of different virus strains, thereby optimizing outcomes in anticipation of future pandemics.

## Introduction

Throughout history, there have been significant impacts on the global economy and public health from infectious diseases, such as the Spanish flu, Middle East respiratory syndrome (MERS), the bubonic plague, and the COVID-19 pandemic. Among measures to control the spread of diseases, lockdown is the most effective, as it can slow down the spread of a virus and potentially prevent a surge in cases and deaths in a short time. However, it can also result in significant economic costs, particularly in terms of lost jobs, reduced economic output, and increased government spending on relief measures [1-4]. Therefore, it is necessary to develop a quantitative method to assess the economic losses and health benefits of strict lockdown measures and their social costs in order to determine which levels of lockdown are sustainable. This will allow us to better prepare for future pandemics.

There has been previous international research on the balance between the health benefits and economic losses of pandemic lockdowns [5,6]. Some studies attempted to correlate the characteristic R0 value (effective transmission number) of virus transmission, as calculated using the SIR (susceptible-infected-recovered) model, with economic behavior [7-10]. Additionally, some studies have used discrete selection experiments to explore the lockdown boundary conditions [11-15]. However, while both methods offer insights into achieving balance, the former only considers the impact of a single parameter, the R0, while the latter is influenced by public fatigue with lockdown when determining the balance boundary. China’s prolonged 3-year lockdown measures during the COVID-19 pandemic resulted in noticeable lockdown fatigue, making it impractical to determine the lockdown boundary conditions through discrete selection experiments.

The World Health Organization (WHO) uses the concept of disability-adjusted life years (DALYs) to quantify the disease burden of various symptoms, whereas lockdown strategies lead to a reduction in individual social and work time, leading to significant productivity losses. Thus, time emerges as a crucial metric for quantifying the impact of lockdowns, with all associated factors being translatable into economic losses by time [16,17]. This allows for the comparison of health benefits and economic losses of various epidemic control policies using time-based or economic metrics. On the other hand, as the virus undergoes evolution and mutation, the comprehensive benefits of lockdown measures will change. Accompanying the reduced virulence of the virus and enhanced human immunity, numerous countries progressively eased their lockdown policies, notably Sweden, which was the first to revoke restrictions, in February 2022. China, the first country to face the COVID-19 epidemic, quickly implemented a city lockdown in Wuhan on January 23, 2020. As of December 7, 2022, China adjusted its dynamic “zero-COVID” policy and no longer implemented lockdowns. Thus, it would be useful to find a quantitative method to determine when and how to implement epidemic control policies.

This study uses an optimization model to estimate the half-lives of the observed case fatality rates (OCFRs) of different strains based on global infection and death data collected by the WHO and strain-sequence time-series data provided by Nextstrain. We establish the connection between the health benefits and economic losses brought by lockdown measures through the calculation of DALYs. Taking China’s city lockdowns as an example, this study determines the cost-benefit boundary of various lockdown measures during the evolution of COVID-19. The main innovations of this research include (1) examining the half-lives of the OCFRs of different strains of the COVID-19 virus, (2) establishing a method for quantifying the economic losses and health benefits of lockdown based on comprehensive calculation of disease burden, and (3) identifying factors influencing the benefits of lockdown. This research will provide an effective tool for the management of COVID-19 and potential future infectious diseases, offering valuable references for government and business decision-making.

## Methods

### Ethical Considerations

The data used in this article were all obtained from a public website, and the study did not involve animal or human experiments, so an ethics committee review statement was not required.

### Data Source

This study sourced the data on the number of SARS-CoV-2 deaths and infections from the WHO [18]. Additionally, temporal evolutionary data on various SARS-CoV-2 strains were acquired from Nextstrain [19].

### Estimation Method for the OCFRs of SARS-CoV-2 Strains

The phenomenon of virus mutation succession is dynamic, evolving over time and potentially leading to the emergence of various mutant strains within a specific timeframe. Throughout this timeframe, the total fatality rate is influenced by the OCFRs of diverse strains, as depicted in equation 1:

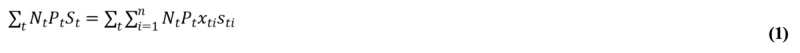



The equation used to calculate the half-life of the OCFR is given below:

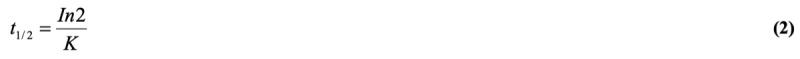



The equation used to calculate the OCFR decay rate constant is as follows:

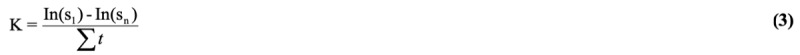



In the above equation, *t* refers to a specific time period, *Nt* refers to the total population at the time period *t*, *Pt* refers to the overall population infection rate at time period *t*, *St* refers to the total OCFR at time period *t*, *n* refers to the number of different strains, *xti* refers to the proportion of the *i*-th strain during time period *t*, and *sti* refers to the OCFR of the *i*-th strain during time period *t*. This parameter can be obtained through multiple linear regression analysis of the OCFR over time, and the general solution to the equation is *St=st*. As the proportion of different strains changed over time, this study selected the overall OCFR of the virus when the strain reached the maximum prevalence rate as the maximum possible observed fatality rate of the strain.

### Calculation of DALYs

Using the classic 2-stage disease model recommended by the WHO, DALYs were used to quantify the disease burden of COVID-19:

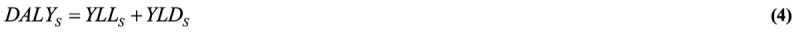

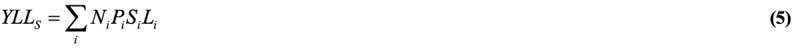

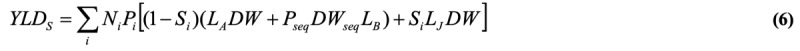

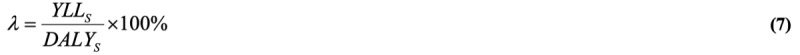









In the above equations, DALYs are per person-year (ppy), life-lost years (YLLs) refer to premature mortality ppy, years lived with disability (YLDs) are given ppy, *i* refers to different age groups (the whole population is divided into 8 age groups: 0-9 y, 10-19 y, 20-29 y, etc, until 70-80 y and >80 y), *N* refers to population number, *P* refers to population infection rate, *S* refers to OCFR, *L* refers to loss of life expectancy caused by death, *DW* refers to disease disability weight, *LA* refers to duration of the disease, *Pseq* refers to probability of sequelae, *DWseq* refers to disability weight of sequelae, *LB* refers to duration of sequelae, and λ refers to the proportion of DALY loss caused by death. Table 1 lists the parameter values.

**Table 1:** The parameters and values used in the calculations.

Parameter	Reference value	Source
Total population (*N*)	1,425,887,360	[20]
Virus infection rate (*P*)	—^a^	—
Observed case fatality rate (*S*)	—	—
Course of disease (years) (*LA*)	0.077	[21]
COVID-19 disability weight (*DW*)	0.051	[21]
Disability weight of sequela (*DWseq*)	0.219	[22]
The incidence of sequelae (*Pseq*)	0.058	[23]
Course of sequelae (years) (*LB*)	0.167	[23]
Lockdown time (days) (*T*)	—	—
Per capita gross national product (dollars per year)	15,308.712	[20]
Control crowd ratio (*a*)	—	—
Productivity weight (ω)	—	[21]

^a^Not applicable.

### Lockdown Economic Calculations

There are 2 types of lockdown methods: blanket lockdown and local or partial lockdown. The former covers a region like an entire province, city, or district to stop many social activities as a whole; the latter covers small-scale entities such as buildings, corridors, and individual families. When an individual lived under lockdown, without mobility or direct contact with the outside world, contact with the virus was effectively avoided. Therefore, the benefit of implementing lockdown measures for a certain period of time was to reduce the population’s disease burden caused by virus infection. The disease burden and economic burden caused by large-scale infection with different strains of SARS-CoV-2 among the population during the epidemic can be calculated using equations 4 and 8. Typically, the disease burden under the condition of total infection or herd immunity can be considered as the possible benefit gained from implementing a lockdown. The difference between a blanket lockdown and local or partial lockdown lies in the scope. For a partial lockdown, the minimum benefit is to prevent the disease burden caused by local population infection from reaching the level of herd immunity, while the maximum possible benefit is to reduce the disease burden within the entire jurisdiction of the government or even globally, as the lockdown can prevent the virus from spreading worldwide. Particularly for highly contagious and fatal viruses such as Ebola and MERS, localized lockdowns can effectively prevent widespread transmission. For a blanket lockdown, the maximum benefit is to ensure that no one in a city becomes infected. Because local governments are responsible for their own economic gains, the product of the gross domestic product (GDP) output per person per year and the loss adjusted per year is used as a measure of the value of disease burden reduction [21,24]:

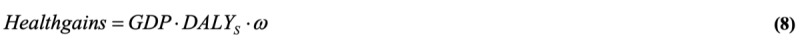



In the above formula, refers to the productivity weight. The values of ω are shown in Table 1.

### Estimated Losses From Lockdown

One of the major losses due to lockdown measures is the decline in GDP resulting from the reduction in social activities and mandatory remote work. According to calculations by the Chinese University of Hong Kong, a 2-week lockdown led to a 31% reduction in GDP for the corresponding month [20]; thus, a 1-month lockdown would be expected to reduce the GDP for that month by approximately 60%. Although lockdown measures have been associated with the exacerbation of chronic diseases and other side effects such as mental illness, this study does not address these aspects due to the lack of sufficient data. Therefore, the loss caused by lockdown can be calculated as follows:

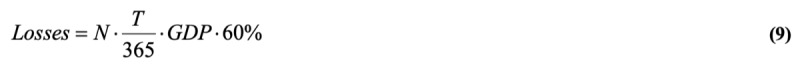



In the above equation, *N* refers to the total population and *T* refers to the number of lockdown days. The values of N and T are shown in Table 1.

### Calculation of the Balance Between Economic Losses and Health Benefits of Blanket Lockdowns

The health benefits of blanket lockdowns can be calculated by the maximum disease burden caused by overall infection or herd immunity (equation 10). The reduction in GDP resulting from the decrease in effective production time is commensurate in value.

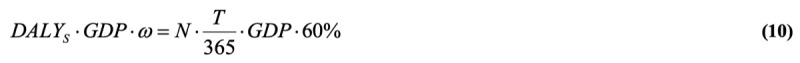

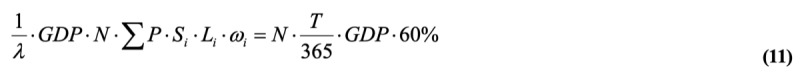





In formulas 9, 10, and 11, *T* refers to the number of lockdown days, *N* refers to the total population under lockdown, GDP exists on both sides of the equation, and *P* refers to the infection rate based on the entire population. Table 1 lists the parameter values.

### Calculation of the Balance Between Economic Losses and Health Benefits of Partial Lockdowns

Similarly, the balance between economic losses and health benefits of a partial lockdown refers to the equivalence between the health benefits resulting from the lockdown and the losses in GDP caused by the lockdown. The cost of the lockdown is primarily influenced by the size of the population affected; that is, there is a certain proportion of individuals in close contact with COVID-19 cases who cease their activities.

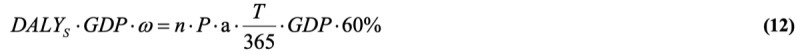

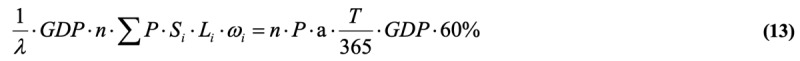





In equations 12 and 13, *a* refers to the number of people quarantined around an infected person. When *a*=1, only the infected person is quarantined. When *a*>1, people who have not been infected may also be restricted from activities. The number of close contacts, subclose contacts, and other companions (not necessarily infected) is determined when a positive infection is found, and there is a redundant cost. *n* refers to the number of people in a community, *P* refers to infection rate, and GDP per capita appears on both sides of the equation. Where life expectancy (*L*), λ, and ω are constants, the benefit and loss balance boundary is inversely proportional to the lockdown time (*T*) and lockdown population ratio (*a*) under the condition that the OCFR (*S*) is relatively stable. Table 1 lists the parameter values.

## Results

### Time Series Trends in OCFRs of Different Strains

**Figure 1:**
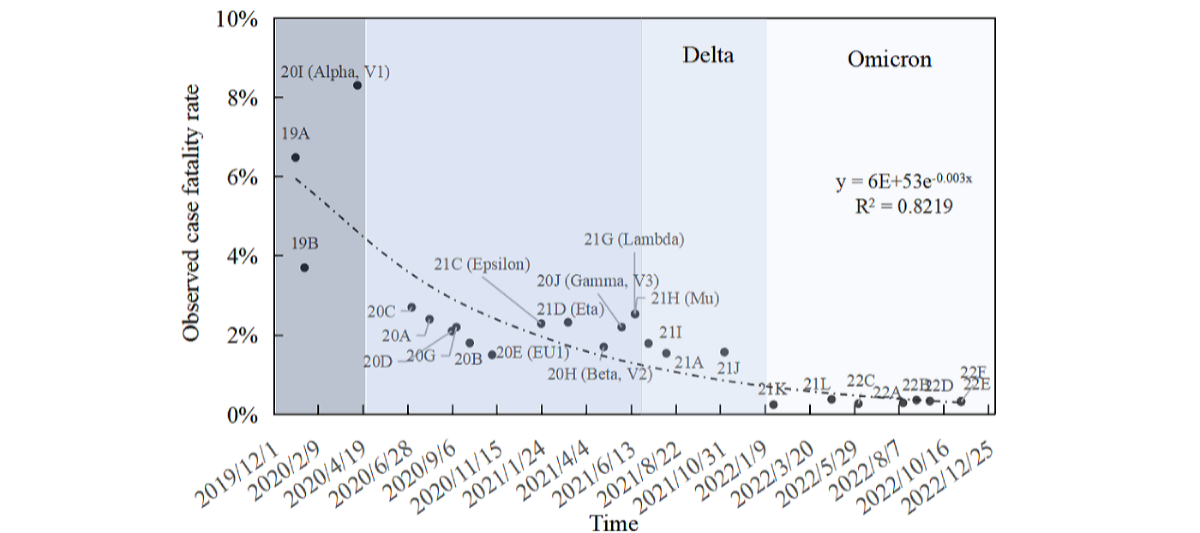
Trends in the observed case fatality rate of SARS-CoV-2.

Based on global mortality data from the WHO since December 2019 and lineage data provided by Nextstrain, Figure 1 illustrates an exponential decline over time in the OCFR of SARS-CoV-2, with a half-life of approximately 8 months. The OCFR for the original SARS-CoV-2 strain was between 3.8% and 8.4%, which then decreased to 2%-3% for the Beta, Gamma, and Lambda variants, and further dropped to 1%-2% for the Delta variant. During the Omicron phase, the OCFR significantly decreased, to about 0.5%. As of January 2023, the OCFR of SARS-CoV-2 in China has been reduced to below 0.1%. According to a report in *The Lancet*, the global OCFR for seasonal influenza from 1999 to 2015 ranged between 0.04% and 0.08%, nearing 0.1% for those older than 75 years [25]. These findings suggest that the OCFR of SARS-CoV-2 is now comparable to that of seasonal influenza. Strains with higher OCFR typically depend significantly on the host’s immune response, leading to reduced reproduction and survival rates, and thus become less competitive compared to less virulent but highly transmissible strains, eventually being replaced over time [26,27].

### Balance Between Economic Losses and Health Benefits With Different Strains in a Blanket Lockdown

In the absence of prevention and control measures, SARS-CoV-2 will continue to infect individuals until herd immunity is achieved. For instance, on December 7, 2022, China adjusted its dynamic zero-COVID policy and no longer implemented lockdowns, leading to concentrated outbreaks of infections. In such a scenario, more than 70% of the population would form an immune barrier after being infected, preventing the virus from spreading continuously [28,29]. Therefore, the maximum loss resulting from complete liberalization is the infection of the entire population. Conversely, the greatest benefit lockdowns can offer to self-sustaining local governments is ensuring that no one within the jurisdiction becomes infected. Different strains have different disease burdens due to their different OCFRs. As the OCFRs of the strains decrease, the disease burden of the population also gradually decreases.

**Figure 2:**
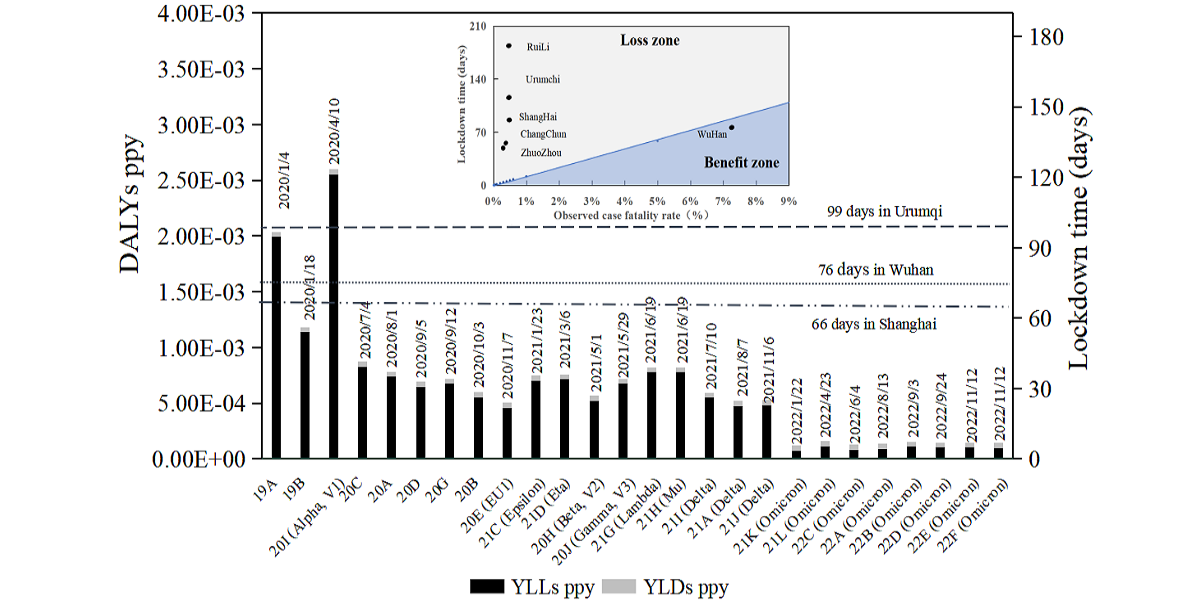
Relationship diagram of lockdown scale and lockdown duration during partial lockdown. DALY: disability-adjusted life year; ppy: per person-year; YLD: year lived with disability; YLL: life-lost year.

Based on the OCFR of each virus strain, the maximum disease loss can be estimated. In Figure 2, the left vertical axis represents the benefits brought by lockdown and the right vertical axis represents the time of the lockdown. Combined with Figure 1, it is clear that the quantitative value of disease losses (measured in DALYs) also declines as the OCFR declines. The maximum benefits from lockdown decrease as the virus strain’s OCFR decreases over time. Using equation 10, the net gain from lockdown can be calculated when health benefits exceed economic losses. For example, during the early lockdown period in Wuhan, involving strains 19A, 19B, and 20I, the health benefits of a 76-day lockdown surpassed the cost line.

The results of the benefit and loss balance calculation indicate that the benefits yielded by the city lockdown strategy are correlated only with the OCFR and the lockdown duration, exhibiting a linear relationship between these 2 factors at the balance point of benefit and loss. As shown in Figure 2 (upper graph), there are 2 distinct zones on either side of the benefit and loss balance line: the benefit zone and the loss zone. When the OCFR is low, a shorter lockdown duration can produce health benefits. Conversely, with a higher OCFR, benefit is achievable even with an extended lockdown. For instance, when the OCFR in Wuhan reached 7% in January 2020, a lockdown period of up to 76 days could still have been beneficial. For the bubonic plague, which has an OCFR of up to 25% [30], a lockdown of more than a year could still produce a strong benefit. However, benefits tend to decline with succeeding virus strains. If the OCFR of a virus strain continues to decline, accompanied by higher false-negative nucleic acid tests, the duration of a lockdown will increase. When the OCFR drops to about 1%, a blanket lockdown is more likely to produce a loss.

### Optimization of Local Lockdown Strategy

**Figure 3:**
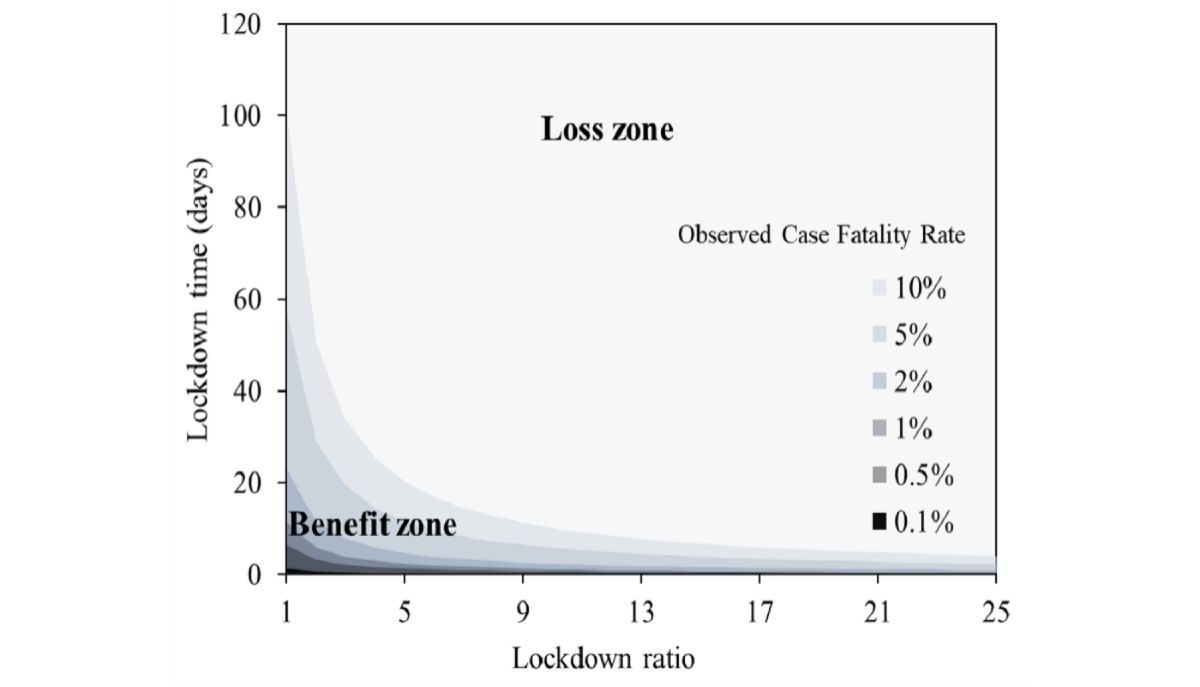
Relationship diagram of lockdown scale and lockdown duration during partial lockdown.

Partial lockdowns have lower cost than blanket lockdowns. According to equation 13, the balance between benefits and losses in a local lockdown is mainly determined by 3 factors: the duration of the lockdown, the size of the covered population, and the OCFR. The benefit from lockdowns primarily comes from reducing the disease losses caused by the epidemic, while losses are mainly due to the reduction in social per capita output, influenced by both the scale and duration of the lockdown. The ratios for different lockdown groups can vary, such as 1:3 (a family of three), 1:5 (5 mixed with 1), 1:10 (10 mixed with 1), 1:20 (20 mixed with 1), 1:40 (a unit), 1:200 (a building), and 1:2000 (a community) for closed management. If benefit calculations only consider the avoidance of infection among isolated individuals, redundant costs arise, as a certain proportion of uninfected individuals in the locked-down area will also waste socially effective labor time. The hyperbolic relationship between population ratio and duration is depicted in Figure 3, in which the upper part of the curve corresponds to losses and the lower left indicates benefits; the darker the color, the greater the likelihood of generating a benefit. When the OCFR is high, the area for benefits (the lower left of the balance line) becomes larger, indicating more flexibility in the scale and duration of the lockdown. When the OCFR is 10% and 1 person in a family of 4 is infected, the maximum lockdown time is 26 days. At this time, in order to reduce the lockdown rate and increase the lockdown benefit, the infected people can be centrally isolated, such as in a shelter. In January 2023, the OCFR for Omicron was less than 0.5% [18], which means that if more than 3 people in close contact were quarantined (with 3 nucleic acid tests mixed with 1), then a state of loss would begin after 2 days, and the quarantine and control measures would be unproductive.

## Discussion

### Principal Results

The management of COVID-19 health emergencies is a huge public challenge, and as of November 28, 2022, the global COVID-19 pandemic had affected a total of 640 million individuals and resulted in 6.63 million fatalities. In China, 9.63 million cases were recorded and 30,000 deaths reported. [20] Remarkably, the lockdown measures implemented in China demonstrated a significant impact, resulting in an estimated reduction of 100 million COVID-19 cases and 1.16 million deaths compared to the global average. China’s approach has thus proved highly effective in the battle against the pandemic and yielded substantial gains. Thus, this paper contributes to documenting and characterizing the history of the pandemic from an epidemiological perspective, refining knowledge about pandemics, enhancing the exploration of the effectiveness of pandemic response measures, establishing the boundary conditions for exiting a strong pandemic response, shortening the emergency transition time of the early response, and providing a knowledge base for designing and framing future pandemic scenarios.

This study discusses 2 types of lockdown strategies: blanket lockdowns and partial lockdowns. In the early stages of a pandemic, when detection levels are low, a blanket lockdown can delay the spread of the epidemic, thereby easing the burden on hospitals and allowing medical institutions time for emergency preparations. When the number of infected people is small and concentrated, improved detection capabilities, such as nucleic acid detection and other methods, can accurately locate and identify infected individuals. Then, a partial lockdown can gradually replace the blanket lockdown. When a person is confirmed to be infected, close contacts (eg, direct contacts) or secondary contacts (eg, indirect contacts) can be identified based on epidemiological findings. The isolation ratio boundary calculated in this study determines which type of contact should be isolated. However, lockdowns based solely on mass nucleic acid screening may not be suitable for all countries. From the perspective of survival and evolution, viruses tend to evolve toward decreased virulence and increased transmissibility. Therefore, with an increasing virus transmission rate and the presence of false negatives in nucleic acid testing, lockdown measures assisted by such screening become largely ineffective, and gatherings for mass nucleic acid testing increase the risk of infection. At this stage, zero transmission is basically impossible to achieve. When herd immunity has not been achieved, long-term, large-scale lockdowns and partial lockdowns are ineffective and only slow the spread of the epidemic, rather than stop it, while imposing a significant economic burden. In this context, sewage virus detection has emerged as a crucial complementary tool. Experience gained from sewage virus monitoring in Hong Kong shows that this method has high sensitivity, reliably detecting 1 positive patient per 20,000 to 40,000 people at a 95% confidence level. Additionally, it can forecast the number of infected individuals 1 to 4 days in advance. Sewage virus detection not only reduces the cost of nucleic acid aggregation testing and the risk of infection but also minimizes the negative impact on productivity and daily life. Each sewage monitoring point can save the effective social time of around 30,000 people who would otherwise be waiting in queues for nucleic acid testing, thus promoting production and restoring normal human life conditions [31]. Furthermore, sewage monitoring holds potential for detecting other types of viruses and can assist in making decisions regarding epidemic prevention by providing an overall and regional assessment of infection levels. With many countries worldwide working toward establishing sewage virus monitoring systems, it is foreseeable that this approach will play a critical role in global epidemic prevention and control in the future.

### Limitations

This study has certain limitations. First, lockdown strategies based on large-scale nucleic acid screening, as implemented in China, may be relatively uncommon in other countries due to factors such as strong government capacity for management and implementation, public cooperation, and the economic ability to bear the associated costs. Consequently, these strategies may not be feasible for all countries and regions. While large-scale nucleic acid screening can effectively and accurately identify infected individuals, false-positive results from nucleic acid tests may undermine the effectiveness of lockdown measures. Furthermore, queues and gatherings of individuals awaiting nucleic acid testing could undermine social distancing efforts and increase the risk of infection.

Second, the calculation of the half-life of the OCFR in this study is based on values derived from GISAID (Global Initiative on Sharing All Influenza Data) statistics rather than the virus’s inherent fatality rate. The OCFR is affected by various factors at different stages of human infection, such as the capacity for detection and screening, active infection (eg, vaccination), and the enhancement of immunity due to repeated passive infections. While the WHO mandates the inclusion of asymptomatic infected individuals in the case fatality rate calculations, the deficiency in early screening capabilities across different countries and regions resulted in an overestimation of the OCFR’s actual impact on mortality. Conversely, the OCFR tends to underestimate the true mortality rate of the strain when there is an improvement in immunity during later stages. Consequently, this introduces a certain degree of uncertainty.

Last, the model is calculated on the basis of short-term economic losses. However, the long-term economic impact of COVID-19 lockdown measures is complex and profound. The economic pressures imposed on society and households by these measures often result in increased drug abuse, mental illness, domestic violence, and even suicide. Given the significant individual variations in these outcomes, our research team is currently tracking the long-term effects of COVID-19. However, we have yet to accumulate long-term cohort tracking data. In future research, we will consider this aspect using more sophisticated models.

### Conclusions

This study shows that with the continuous enhancement of detection capabilities and the continuous formation of population immunity, the OCFR of COVID-19 decreases exponentially, and the half-life is about 8 months. In the infectious disease lockdown strategy model, there is a close correlation between the benefits of lockdown and OCFR. During the early transmission period of SARS-CoV-2, especially under conditions of high OCFR, the lockdown strategy in Wuhan brought huge health benefits to the population. However, as the OCFR decreases, these health benefits gradually diminish and eventually transition into losses. Although large-scale lockdown strategies may not be universally applicable for political reasons, they appear to be the least disruptive option in the early stages of an outbreak. The partial lockdown model mentioned in this study can provide a reference for various countries and regions. This study establishes a connection between saving lives and protecting the economy, indicating that these 2 objectives are not opposites.

This study provides a critical scientific basis for policy makers, guiding the appropriate timing for the implementation or relaxation of lockdown measures. It offers essential insights for formulating public health management strategies related to COVID-19 and future pandemics, providing indispensable reference information for governments and decision-making entities. By integrating thorough risk assessments with economic impact analyses, our research aims to develop more effective and compassionate approaches to pandemic response, with dual objectives: to protect public health and safety and to minimize negative impacts on the socioeconomic landscape.

## Abbreviations

DALY: disability-adjusted life year
GDP: gross domestic product
GISAID: Global Initiative on Sharing All Influenza Data
MERS: Middle East respiratory syndrome
OCFR: observed case fatality rate
ppy: per person-year
SIR: susceptible-infected-recovered
WHO: World Health Organization
YLD: year lived with disability
YLL: life-lost year
